# Xiang Qin Kang Gan Granules Treated the Human Coronavirus 229E Induced Pneumonia with Damp-Heat Syndrome in Mice

**DOI:** 10.1155/2022/7609550

**Published:** 2022-09-23

**Authors:** Fang Cao, Zhaoheng Liu, Qingxun Hao, Ronghua Zhao, Lei Bao, Xiaolan Cui, Yang Jiao

**Affiliations:** ^1^Dongfang Hospital Affiliated to Beijing University of Chinese Medicine, No. 6 Fang Zhuang Fang Xing Yuan, Fengtai District, Beijing 100078, China; ^2^School of Life Sciences, Beijing University of Chinese Medicine, Northeast Corner of the Intersection of Yang Guang Nan Da Jie and Bai YangDongLu, Fangshan District, Beijing 102488, China; ^3^Beijing University of Chine se Medicine, No. 11 Bei San Huan Dong Lu, Chaoyang District, Beijing 100029, China; ^4^Institute of Chinese Materia Medica, China Academy of Chinese Medical Sciences, Beijing 100025, China

## Abstract

Coronavirus disease 2019 (COVID-19), which causes severe respiratory illness, was first reported in Wuhan, China. The etiology of the disease is a new novel coronavirus named severe acute respiratory syndrome coronavirus 2 (SARS-CoV-2), which was reported to share the same origin as SARS-CoV, causing severe public health events in 2002. Unlike the SARS-CoV, which was conquered in the early summer of 2003, this virus was still contagious widely and reached a pandemic level. It can still spread fast even if the season's temperature is raised. Here, we made a model of pneumonia of human coronavirus 229E (HCoV-229E) with damp-heat syndrome treated by Xiangqin Kanggan granules to find a new medicine for treating these kinds of infectious diseases coronaviruses induced.

## 1. Introduction

COVID-19 has been dramatically spreading worldwide, and until now, there has been no specific medicine for this disease. Traditional Chinese medicine (TCM) has played an important role in the fight against this new virus, and it is widely used in China and some other countries.

The efforts of TCM are especially helpful in relieving syndromes. It is reported that in the COVID-19 treatment, compared with the use of western medicine alone, patients treated with TCM reduced fever, improved shortness of breath, relieved cough, and stabilized blood oxygen saturation. It also can relieve symptoms such as, anorexia, and fatigue.

Some of the Chinese medicine masters have discussed the syndrome of COVID-19 according to the syndrome differentiation of the Chinese medicine system, since most patients have fever, diarrhea, and yellow phlegm, so the disease can be defined as a damp-heat syndrome [1]. Xiang Qin Kang Gan Granules (XQKGGs) were used to treat the damp-heat syndrome in respiratory disease in Dongfang Hospital, which is an affiliated hospital with the Beijing University of Chinese Medicine. From clinical observation, it has shown efficacy and safety, but the data has not yet been published.

In this study, a mouse model of the damp-heat syndrome was prepared, and mice were infected with human coronavirus 229E to detect the lung index, virus replication, cytokines of cAMP and prostaglandin E2 (PGE2) in the hypothalamus, cytokines of interleukin 1 (IL-1*β*), CD14, malondialdehyde (MDA) and superoxide dismutase (SOD) in lung tissue, as well as the comparison of pathology in the groups of sham, damp-heat, 229E, model, chloroquine phosphate, and XQKGG (low, middle, and high dose).

## 2. Material and Method

### 2.1. Reagents and Material

Mouse IL-1*β*ELISA Kit (lot number 200701266), mouse CD14 ELISA Kit (lot number 200514055), mouse SOD ELISA Kit (lot number 200708682), and mouse MDA ELISA Kit (lot number 200625030) were purchased from bio-techne. Mouse PGE2 ELISA Kit (lot number 200604923) and mouse cAMP ELISA Kit (lot number 200706528) were purchased from Shanghai Enzyme-linked Bio-Tech Co., Ltd. Human Coronavirus (HCoV-229E) Real Time RT-PCR Kit (lot number P20191201) was purchased from Shanghai Liferiver Bio-Tech Co., Ltd.

### 2.2. Preparation of HCoV-229E

200 *μ*l of HCoV-229E virus solution was added to a single layer in a culture flask with a single layer of raw 264.7 cells and placed the flask in a 37°C 5% CO_2_ incubator. When 80% of the cells show a cytopathic effect (CPE), place the cell culture flask at −80°C after repeated freezing and thawing of the virus solution 3 times, and this is used for testing virus virulence.

A HCoV-229E virus solution was diluted in different titers from 10^−1^–10^−8^, 100 *μ*l per well, with 4 replicate wells for each concentration, and a normal cell control was also set. Then, it was put in a 37°C 5% CO_2_ incubator. The 50% cytopathic concentration (TCID50) was recorded according to Reed–Muench after 72–96 hours.

### 2.3. Preparation of XQKGG

XQKGG was purchased from Beijing Kang Ren Tang, the Pharmaceutical Industry Co., Ltd. Its components are Herba Pogostemonis, Radix Scutellariae, Semen Armeniacae Amarum, Cortex Magnoliae Officinalis, and Rhizoma Belamcandae, and its total net weight is 54 g.

### 2.4. Animal Model and Experiment Design

64 Balb/c male mice were purchased from Beijing Vital River Laboratory Animal Technology Co., Ltd. After 7 days of adaptive feeding, the model was made. All mice were randomly assigned to the sham group, damp-heat group, 229E group, model group, chloroquine phosphate group, and XQKGG group (high, middle, and low dose). In all groups except the sham group and 229E group, the mice were put into and kept in an artificial climate chamber with 95 ± 5% relative humidity, no wind, and a temperature of 37 ± 2°C 4 hours per day for one week. On the 5^th^ day, the mice were anesthetized by ether, and the mice were infected with 100TCID50 HCOV-229E virus drop (50 *μ*l) into the nose. This day was marked as the 1^st^ day, we gave chloroquine phosphate (0.09 g/kg·bw) to the chloroquine phosphate group, and XQKGG (0.002 ml/kg·bw) to the XQKGG group. The concentration ratio was 4 : 2 : 1. The middle dose was converted according to the dosage in humans. Meanwhile, the other groups were fed with the same volume of saline. The gavage lasted for 3 days. On the 4^th^ day, all the mice were euthanized by exsanguination after the injection of 1% pentobarbital (70 mg/kg·bw) for laboratory detection.

The mice were bred in the Biosafety P2+ laboratory of the Institute of Chinese Materia Medica, China Academy of Chinese Medical Sciences. The animal experiment operation complies with the regulations of the National Institutes of Health (NIH) and the Beijing Experimental Animal Ethics Committee and is approved by the Animal Ethics Committee of the Institute of Chinese Materia Medica, China Academy of Chinese Medical Sciences.

### 2.5. Sample Collection

The blood was collected, and the lung index and inhibition rate were calculated after measuring the lungs weight using the formula as follows:

(1) $$\begin{array}{c} \text{Lung index}=\left[\frac{\text{wet lung weight}\left(g\right)}{\text{body weight}\left(g\right)}\right]\times 100\%,\\ \text{Lung index inhibition rate}=\frac{\left(\text{model group lung indexadministration group lung index}\right)}{\left(\text{Model group lung indexcontrol group lung index}\right)}\times 100\%. \end{array}$$

After the weight measure, 3 of the mice's lungs in each group were fixed by polyoxymethylene for HE staining, while the other lungs were stored in the −80°C refrigerator for RT-PCR.

The hypothalamus tissues were taken and stored at −4°C. 3 mg of hypothalamic tissue and 150 *μ*L of physiological saline were mixed and homogenized by an ultrasonic cell disruptor. The samples were centrifuged with −4°C 1000 r/s for 10 minutes. After aspirating the supernatant, the samples were aliquoted and stored at −80°C for test.

### 2.6. General Histological Staining

The lungs were fixed for more than 24 h, then dehydrated and embedded in paraffin and cut into 5 *μ*m sections. The sections were (1) putintoxylol III, each for 15 minutes; (2) put into the ethanol with the concentration of 100% I, II ⟶ 95% I, II ⟶ 90% ⟶ 80% ⟶ 70% ⟶ 60% ⟶ 50%, each for 2 minutes and washed with running water for 5 minutes; (3) stained with hematoxylin for 15 minutes and washed the extra hematoxylin; (4) put into 1% hydrochloric acid alcohol for 30 sand washed with running water for 10 minutes; (5) stained with eosin for 15 minutes and washed for 1 minute, (6) put into the ethanol with the concentrations of50% ⟶ 60% ⟶ 70% ⟶ 80% ⟶ 90% ⟶ 95% I, II ⟶ 100% I, II, each for 2 minutes; and (7) put into xylol I, II, each for 15 minutes.

### 2.7. RT-PCR

The lung tissue was used for this test. The reaction tube was put through RT-PCR and the cycle parameters were set as 45°C × 10 minutes, 95°C × 15 minutes, and then 95°C × 15 s ⟶ 60°C × 60 s for 40 cycles, with single-point fluorescence detection at 60°C. Fluorescence channel detection options include FAM and HEX/VIC/JOE channels.

### 2.8. Flow Cytometry

3 drops of blood (about 150 *μ*l) were added to a centrifuge tube with 10 ml PBS, centrifuged at 1600 rpm for 5 minutes, the supernatant was discarded and added 1 ml of blood red cell lysing reagent for about 5–10 minutes. Then, 10 ml PBS was added to stop the lysis, centrifuged at 2000 r/s for 5 minutes at 4°C, and the supernatant was discarded. The resuspended cell pellet was resuspended in 10 ml PBS and centrifuged at 2000 r/s for 5 minutes at 4°C, the supernatant was discarded again. 200 *μ*l block buffer was added and the cell was transferred to a 1.5 ml ep tube and sealed at 4°C for 30 minutes. The cell suspension was centrifuged at 2000 r/s for 5 minutes at 4°C, and the supernatant was discarded. The flow cytometry antibody was added, and it was stained for 30 minutes at 4°C; 1 ml PBS was added, and the tube was centrifuged at 2000 r/s for 5 minutes at 4°C; and then the supernatant was discarded. The samples were transferred to a flow tube and tested with flow cytometry (DxFLEX, Beckman, USA).

### 2.9. Cytokine Measurement Using ELISA

Levels of PGE2, cAMP, IL-1*β*, CD14, MDA, and SOD were measured with the ELISA kits. All procedures were performed in accordance with the manufacturer's instructions.

2.9 All graphing and statistical analyses were performed using SPSS 16.0. If the data met a normal distribution, the results were shown as mean ± standard deviation (mean ± S. D); otherwise, the results were shown as median (interquartile range, P75–P25). Comparisons among multiple groups were analyzed with one-way ANOVA. Single comparisons were made with the LSD test. If the data did not meet a normal distribution, the results were analyzed with the Kruskal–Wallis rank sum test. *P* values ≤ 0.05 were considered statistically significant.

## 3. Results

### 3.1. XQKGG Inhibits Viral Replication in Coronavirus 229E Induced Pneumonia

Compared with the sham group, the lung index of the model significantly increased (*P* < 0.01). Compared with the model group, the lung indices of all three doses of XQKGG groups were reduced. The low dose group could significantly reduce the lung index when compared with the model group (*P* < 0.05). The lung index inhibition rates of high, middle, and low doses are 20.66%, 26.50%, and 34.90, respectively (Table 1).

### 3.2. XQKGG Ameliorates Coronavirus 225E Induced Lung Injury

**Figure 1:**
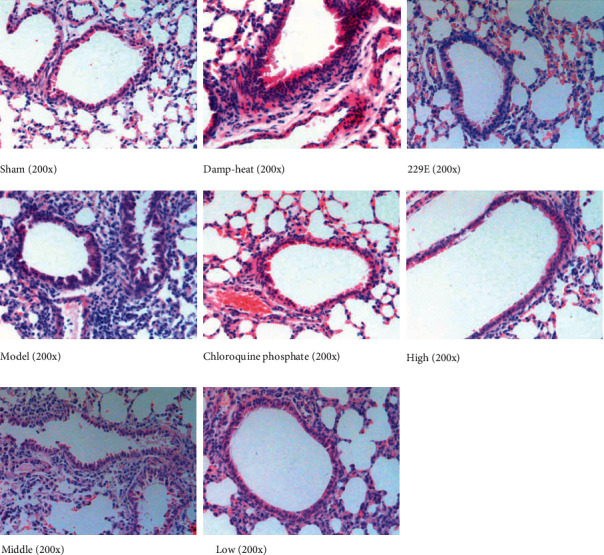
Effect of XQKGG on HCoV-229E induced pulmonary histopathology in mice. Pulmonary structure and tissue of bronchiole and pulmonary interstitial inflammatory infiltration in each group. There was less inflammation and structure destroyed in XQKGG groups.

HE staining was performed to evaluate pathological changes in lung tissues. In the damp-heat group and 229E group, the inflammatory cells like lymphocytes were infiltrated, histocytes were enlarged, and the interstitial mucus was thickened. Meanwhile, the intima of the bronchioles was swollen, with an increase of intimal cells, a small amount of inflammation and exudation, and a significant increase in smooth muscle tissue cells. In the model group, there were more inflammatory cells when compared with the damp-heat group, the inflammatory cells were clustered together, and the damage to the lung tissues was more severe than that in the damp-heat group. And there were more red blood cells and mucus exuded in the interstitium. Compared with the groups, the chloroquine phosphate group and the 3 XQKGG groups showed that the inflammatory cell infiltration and bronchioles injury were improved (Figure 1).

### 3.3. Effect of XQKGG on Levels of Virus in Lung Tissue

There was plenty of expression of 225E nucleic acid in the model group, and there was no 225E nucleic acid found in the sham group. The level of the acid in the 2 XQKGG groups (middle dose and low dose) was lower with statistical significance compared with the model group (*P* < 0.05, *P* < 0.01). (Table 2).

### 3.4. XQKGG Inhibits the Immune Reaction in Blood

Compared with the sham group, the percentage of B cells in the blood of the model group had the trend to increase (*P* > 0.05), and the high and middle doses of XQKGG groups could significantly reduce the percentage of B cells when compared with the model group (*P* < 0.05, *P* < 0.01). (Table 3).

### 3.5. Effect of XQKGG on Levels of PGE2 and cAMP in the Hypothalamus

Compared with the sham group, the content of cAMP in the hypothalamus of mice in the model group was significantly increased (*P* < 0.01), but there was no statistical difference in PGE2 between the sham group and the model group. (*P* > 0.05). Compared with the model group, the content of cAMP in 3 XQKGG was significantly decreased (high dose *P* < 0.05, middle and low dose *P* < 0.01) (Table 4).

### 3.6. Effect of XQKGG on Levels of IL-1*β*, CD14, MDA, and SOD in Lung Tissue

**Figure 2:**
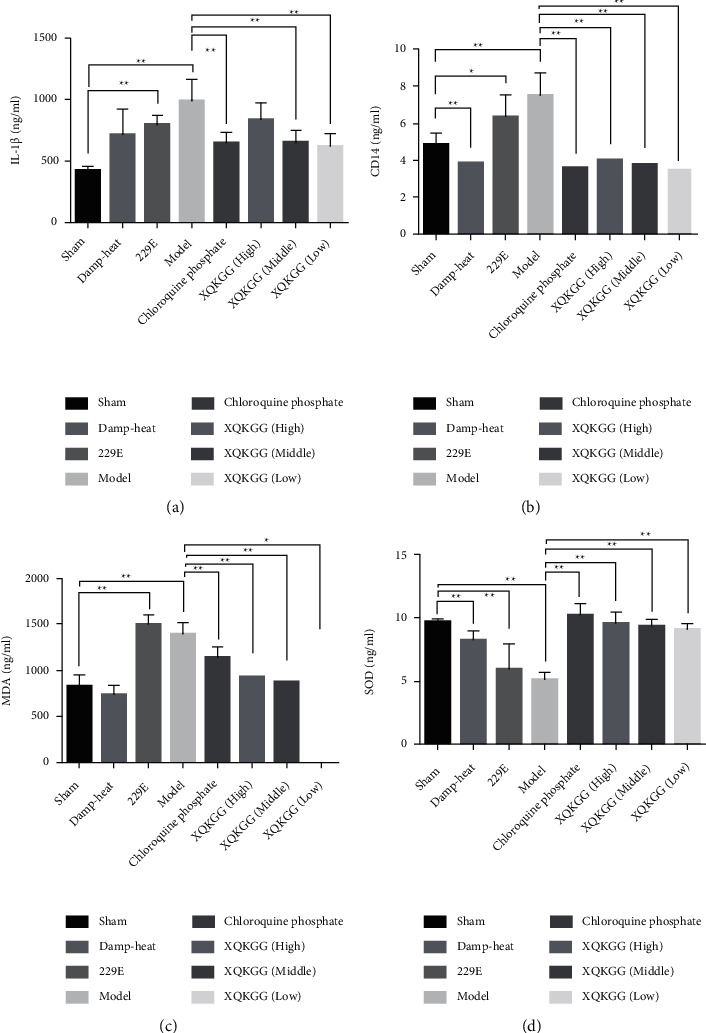
Comparison of the cytokines of IL-1*β* (a), CD14 (b), MDA (c), and SOD (d) in rat lung tissue with the method of ELISA. Comparison between the sham group and the 229E group with a level of IL-1*β* (*P* < 0.01), CD14 (*P* < 0.05), MDA (*P* < 0.01), and SOD (*P* < 0.01); comparison between the sham group and the model group with a level of IL-1*β* (*P* < 0.01), CD14 (*P* < 0.01), MDA (*P* < 0.01), and SOD (*P* < 0.01); comparison between the model group and the chloroquine phosphate group/high and middle doses of XQKGG groups with the level of IL-1*β* (*P* < 0.01), CD14 (*P* < 0.01), MDA (*P* < 0.01), and SOD (*P* < 0.01); comparison between the model group and a low dose of XQKGG group with a level of IL-1*β* (*P* < 0.05), CD14 (*P* < 0.01), MDA (*P* < 0.05), and SOD (*P* < 0.01).

Compared with the sham group, the contents of IL-1*β*, CD14, and MDA in the lung tissue in the model group were significantly increased (*P* < 0.01), and the contents of CD14 and MDA in three doses of the XQKGG groups were significantly decreased (3 doses of CD14 *P* < 0.01, high dose and middle dose of MDA *P* < 0.01, and low dose of MDA *P* < 0.05), and the content of SOD was significantly increased (*P* < 0.05) The middle and low doses of the XQKGG groups could significantly reduce the content of IL-1*β* when compared with the model group(*P* < 0.01). (Figure 2).

## 4. Discussion

To imitate the syndromes of patients with COVID-19-induced pneumonia, this experiment established a new type of model by infecting mice with the 229E virus in a damp-heat environment. Meanwhile, the model could be used for drug research.

We could find that food intake and water consumption were reduced, and the coat color was dull, loose, and rough. The stool became soft. Additionally, the model mice were surely weak with fever and slow to respond. From the test results, the lung virus nucleic acid test is positive, and the inflammatory response indicators are significantly increased. Obvious pathological changes occurred in the tissues. Therefore, the model was typical damp-heat syndrome pneumonia. After administration of XQKGG, the symptoms of the above were significantly improved.

XQKGG is composed of Herba Pogostemonis, Radix Scutellariae, Semen Armeniacae Amarum, Cortex Magnoliae Officinalis, and Rhizoma Belamcandae. This medicine is based on the theory of Chinese medicine symptom-differentiation treatment. Since there are still no specific effective drugs for COVID-19, symptomatic treatment and life support are essential. The XQKGG can relieve the symptoms, and the results show the effectiveness of the antivirus and explain some of the mechanisms. Also, from the pharmacological studies, we found evidence that XQKGG could treat the disease.

Pharmacological studies have shown that Herba Pogostemonis contains monoterpene, sesquiterpene, and micromolecular alcohol with the functions of antivirus, antioxidation, anti-inflammatory, analgesic activities, and intestinal barrier function protection [2].

Radix Scutellariae contains baicalein, baicalin, and wogonin [3], which can improve the inflammation in acute lung injury and gingivitis [4, 5]. Furthermore, Zhang et al. [6] proved that baicalein can significantly inhibit the expression of proteins of iNOS and COX-2 in vitro, so it has the function of antioxidative stress.

Semen Armeniacae Amarum can be used to treat cough and asthma. The main ingredient is amygdalin, which has the functions of an expectorant and an antitussive [7].

The most effective component of Cortex Magnoliae Officinalis is quercetin. It has anti-inflammatory, antiviral, antitumor, hypoglycemic, and immune regulation effects [8].

The main phytochemicals of Rhizoma Belamcandae are isoflavones, xanthone glycosides, stilbenes, simple phenols, and quinones. Pharmacological studies have shown the herb has the functions of antimutagenic, anti-inflammatory, antiangiogenic, and hypoglycemic, and it is mainly used in respiratory diseases [9].

In this study, the lung index, the level of virus in the lungs, and lung pathology were tested, and the results showed that XQKGG could reduce the inflammatory lung injury as well as the level of virus. In addition, the high and middle doses of XQKGG could significantly reduce the percentage of B cells when compared with the model group.

B cell response can adaptively protect the body against viruses. After the primary infection, it can supply neutralizing antibodies against reinfecting viruses [10]. However, the high response may cause immune injury in the target organ. The high and middle doses of XQKGG could lower the percentage of B cells, which suggested that the medicine could lower the virus content and protect the organ from overimmune injury. The result of the levels of the virus also showed that the process presumed that.

We also detected the levels of cytokines of PGE2 and cAMP in the hypothalamus to explore the mechanism of the disease and the effect of XQKGG. From the result, we found that the groups of XQKGG could significantly lower the cAMP in the hypothalamus, and the XQKGG had the trend to decrease the PGE 2.

Hypothalamus is the temperature regulation center of the body, the anterior part of the hypothalamus is the location of temperature-sensitive neurons, and the posterior part of the hypothalamus is an integrated part of body temperature regulation, which can adjust the body's heat production and dissipation process to keep the body temperature at a certain level.

Fever is a self-defense response of the body and is a symptom shared by infectious diseases and inflammation [11, 12]. The induction and maintenance of fever during infection involve coordination and interaction between the immune and nervous systems. Immune perception of infection starts with pathogen-related molecular patterns such as virusRNA with pathogen recognition receptor (PRR), which is expressed by the innate immune cell populations, including macrophages and neutrophils [13]. These immune cells release PGE2 as well as other cytokines (interleukin-1 (IL-1), IL-6, etc.) which induce fever. IL-6 can make IL-6 in the probiotic nucleus of the hypothalamus to induce cyclooxygenase 2(COX2), and this enzyme can produce additional PGE2 [14, 15].

cAMP is another central pyrogenic medium that has been extensively studied. It acts on PO/AH temperature-sensitive neurons, changes the discharge frequency, and increases the set point of body temperature.

There are mainly three heating signal pathways that are recognized. They are converged to the junction of PGE2 when they are activated. Then, with the change of the body's PGE2 synthesis, the EP3 signal pathway is activated, which causes fever. When the activation of fever pathways leads to a change in the content of PGE2, it binds to the transmembrane receptor EP3 on the cell membrane and couples to the G protein, which then activates the macromolecular protein AC, which activates ATP in the cell that is activated to generate cAMP [16].

From the result of ELISA, XQKGG could reduce the level of IL-1*β*, CD14, MDA, and increase the level of SOD.

IL-1*β* plays an important role in the early stages of pulmonary inflammatory diseases. In acute lung injury, IL-1*β* is produced earlier and can induce and amplify inflammatory reactions. It also can judge the degree of lung injury [17].

CD14 is widely discussed in microbial infections, and it plays an important role in innate immunity. In addition, it is indispensable in inflammatory disorders. For example, it promoted LPS-induced inflammation in multiorgan injuries and disorders [18].

Oxidative stress is produced by free radicals and is considered as an essential factor leading to apoptosis, aging, and other diseases [19–21]. Virus infection has a close relationship with oxidative injury; it causes oxidative stress and intensifies the pathological process.

MDA is the most important product of animal cell membrane lipid peroxidation. The aggravation of cell membrane damage is also caused by MDA. MDA content determination is a commonly used indicator in physiological research [22].

SOD is an enzyme that can protect the body from oxidative stress damage and can catalyze the disproportionation of superoxide anion radicals to produce water and hydrogen peroxide, which can effectively remove free radicals in the human body. It can repair tissue damage or slow down the formation of inflammation. It also has a positive effect on protecting cells from further damage [22].

Therefore, XQKGG may lower cAMP and PGE2 in the hypothalamus and downregulate IL-1*β*, CD14, MDA, and upregulate SOD to protect the body from lung injury caused by coronavirus 229E and heat-dampness in mice.

In addition, chloroquine phosphate was used as an appositive control drug, and we found it has a similar function to XQKGG. In this way, the XQKGG compared with chloroquine phosphate can demonstrate effectiveness. Furthermore, it may help us find another way to treat the infectious disease.

## 5. Conclusions

We noted that XQKGG could reduce the injury of pneumonia induced by coronavirus 229E and damp-heat environment in mice, including reducing levels of virus in lung tissue, which indicated that XQKGG have the antivirus function. Meanwhile, pathological injury of the lungs was alleviated, showing that XQKGG could protect the lungs from injury. XQKGG also inhibited the immune reaction in the blood, lowered cAMP and PGE2 in the hypothalamus, and downregulated the IL-1*β*, CD14, MDA, and upregulated the SOD, telling the mechanism of the medicine to protect the mice from injury through inhibiting inflammation and oxidative damage as well.

## Figures and Tables

**Table 1:** Comparison of the lung index in groups.

Group		Dose(g/kg/d)	Quantities	Lung index	Lung index inhibition rate
				(Lungs weight *∗* 100/bw)	%
Sham		—	10	0.70 ± 0.08	—
Damp-heat		—	10	0.73 ± 0.06	—
229E		—	10	0.96 ± 0.13^##^	—
Model		—	10	0.94 ± 0.07^##^	—
Chloroquine phosphate		0.09	10	0.85 ± 0.14	34.63
XQKGG	High	—	10	0.89 ± 0.06	20.66
	Middle	—	10	0.87 ± 0.09	26.50
	Low		10	0.85 ± 0.09^*∗*^	34.90

Compared with the sham group, ^##^*p* < 0.01; compared with the model group, ^*∗*^*p* < 0.05.

**Table 2:** Comparison of the levels of virus in lung tissue in groups (*n* = 6).

Group		Level of 225E nucleic acid (copies/ml)
Sham		0
Damp-heat		0
229E		12336.04 ± 3262.06^##^
Model		16449.05 ± 5725.45^##^
Chloroquine phosphate		6590.84 ± 2196.45^*∗∗*^
XQKGG	High	34835.12 ± 49849.39
	Middle	8519.02 ± 2031.83^*∗*^
	Low	6173.94 ± 2220.10^*∗∗*^

Compared with the sham group, ^##^*p* < 0.01; compared with the model group, ^*∗*^*P* < 0.05, ^*∗∗*^*P* < 0.01.

**Table 3:** Comparison of the percentages of immune cells in the blood in groups (*n* = 6).

Group		Dose (g/kg/d)	Percentages of B cells (%)
Sham		—	25.33 ± 5.09
Damp-heat		—	20.57 ± 4.06
229E		—	16.64 ± 7.21^#^
Model		—	28.09 ± 4.37
Chloroquine phosphate		0.09	15.05 ± 4.83^*∗∗*^
XQKGG	High	—	12.33 ± 6.48^*∗∗*^
	Middle	—	19.71 ± 6.47^*∗*^
	Low	—	21.86 ± 6.32

Compared with the sham group, ^##^*p* < 0.01^#^*p* < 0.05; compared with the model group, ^*∗*^*P* < 0.05, ^*∗∗*^*P* < 0.01.

**Table 4:** Comparison of cytokines in hypothalamus tissue of mice in groups (*n* = 6).

Group		Dose (g/kg/d)	Levels of cytokines (ng/ml)	
			PGE2	CAMP
Sham		—	554.72 ± 49.06	7121.33 ± 718.93
Damp-heat		—	571.39 ± 93.63	6368.00 ± 800.87
229E		—	642.50 ± 41.12^##^	13431.33 ± 1096.44^##^
Model		—	654.72 ± 110.48	11988.00 ± 1283.92^##^
Chloroquine phosphate		0.09	628.33 ± 75.15	10088.00 ± 484.77^*∗∗*^
XQKGG	High	—	757.78 ± 57.76	10324.67 ± 841.68^*∗*^
	Middle	—	690.28 ± 64.79	9631.33 ± 507.96^*∗∗*^
	Low	—	748.33 ± 108.18	9711.33 ± 713.83^*∗∗*^

Compared with the sham group, ^##^*p* < 0.01; compared with the model group, ^*∗*^*P* < 0.05, ^*∗∗*^*P* < 0.01.

## Data Availability

The data used to support the findings of this study are available from the corresponding author upon request.
